# Investigation of Brain Functions with Fluorescence Imaging Techniques

**DOI:** 10.14789/jmj.JMJ21-0051-OT

**Published:** 2022-04-15

**Authors:** YOHEI OKUBO

**Affiliations:** 1 Department of Cellular and Molecular Pharmacology, Juntendo University Graduate School of Medicine; 1 Department of Cellular and Molecular Pharmacology, Juntendo University Graduate School of Medicine

**Keywords:** calcium, fluorescence microscope, glutamate, inositol 1,4,5-trisphosphate, metabotropic glutamate receptor

## Abstract

An intricate interplay of complex spatio-temporal events underlies brain functions. Therefore, clarifying these dynamic processes is indispensable for revealing the mechanisms of brain functions. Fluorescence imaging is a powerful technique for visualizing cellular and molecular dynamics in the brain. Recent developments in fluorescent indicators and specialized optics have advanced research in the field of neuroscience. In this review, I will exemplify the power and beauty of fluorescence imaging by discussing my work focusing on the molecular dynamics of metabotropic glutamate receptor (mGluR) signaling at the synapse. By developing novel fluorescent indicators for glutamate, inositol 1,4,5-trisphosphate and Ca^2+^ within the endoplasmic reticulum, I succeeded in imaging the spatio-temporal dynamics of synaptic mGluR signaling, which led to the identification of novel mechanisms of mGluR-mediated glutamatergic neurotransmission. These discoveries highlight the importance of the development and application of novel fluorescence imaging techniques for the investigation of brain functions.

## Introduction

Complex spatio-temporal processes are the foundation of the information-processing power of the brain. For example, the dynamic interplay of different brain regions is the basis of the regulation of physical and mental states. Spatio-temporal firing patterns of neuronal networks in the brain encode sensory inputs and behavioral outputs. Elaborate molecular dynamics at synapses is essential for synaptic transmission, the elemental basis of brain functions. Therefore, the visualization of spatio-temporal dynamics is key to understanding the mechanisms of brain functions. Fluorescence microscopy is a powerful technique for imaging cellular and molecular dynamics in living samples. Various fluorescent molecules including small-molecule fluorophores and fluorescent proteins have been used as biocompatible dyes for specific labeling of molecules of interest. Furthermore, fluorescent molecules have been functionalized as fluorescent indicators for imaging the dynamics of various events such as local Ca^2+^ signaling at synapses^1)^. Imaging within the intact brain tissue is crucial to the study of brain functions. However, light scattering by brain tissue is a major obstacle for conventional fluorescence microscopy. Development of two-photon microscopy^2)^ solved this problem and allowed high-resolution imaging deep within the brain tissue *in vivo*^3)^. Recent progress in fluorescent indicators and optics have revolutionized the neurosciences by enabling long-awaited analyses such as the imaging of synaptic plasticity at single-synapse resolution^4, 5)^, the spatio-temporal analysis of firing patterns of hundreds of neurons *in vivo*^6)^, the identification of circuit rewiring upon learning^7)^, and the direct imaging of neurotransmitter dynamics^8, 9)^. The continuous development of fluorescence imaging techniques has been advancing research into brain functions.

## Fluorescence imaging of metabotropic glutamate receptor (mGluR) signaling

In this review, I will discuss fluorescence imaging of brain functions with examples from my studies. My work has focused on the molecular dynamics of mGluR signaling at synapses^10)^. Glutamatergic transmission is mediated not only by ionotropic glutamate receptors, but also by G protein-coupled mGluRs. There are eight types of mGluRs divided into group I (mGluR_1_ and mGluR_5_), group II (mGluR_2_ and mGluR_3_) and group III (mGluR_4_, mGluR_6_, mGluR_7_ and mGluR_8_). Group II and III mGluRs are mainly distributed at presynaptic terminals and involved in the inhibition of neurotransmitter release via G_i/o_ protein signaling. On the other hand, group I mGluRs are postsynaptic receptors that mediate the production of inositol 1,4,5-trisphosphate (IP_3_) via G_q_ protein to induce Ca^2+^ release from the endoplasmic reticulum (ER) via the IP_3_ receptor. This postsynaptic mGluR-IP_3_-Ca^2+^ signaling is involved in many important synaptic functions including synaptic plasticity. Furthermore, group I mGluR- targeted drugs exert various psychiatric effects. Although the functional significance of group I mGluRs is obvious, mGluR-targeted therapeutics has not been available so far. This is mainly due to a lack of knowledge about the detailed mechanism of synaptic mGluR signaling. To address this issue, the investigation of the dynamics of molecular signaling *in situ* is essential. Therefore, I set out to develop techniques for the direct imaging of glutamate^9, 11)^, IP_3_^12-14)^ and Ca^2+^ within the ER^15^ at synapses in brain tissue.

## Clustered input-dependent glutamate spillover

mGluRs are localized to the perisynaptic regions that border the synaptic clefts. Therefore, glutamate spillover from the synaptic clefts is required for the activation of mGluRs. However, the characteristics of the glutamate spillover were unclear. Therefore, I decided to directly analyze glutamate spillover by glutamate imaging.

**Figure 1:**
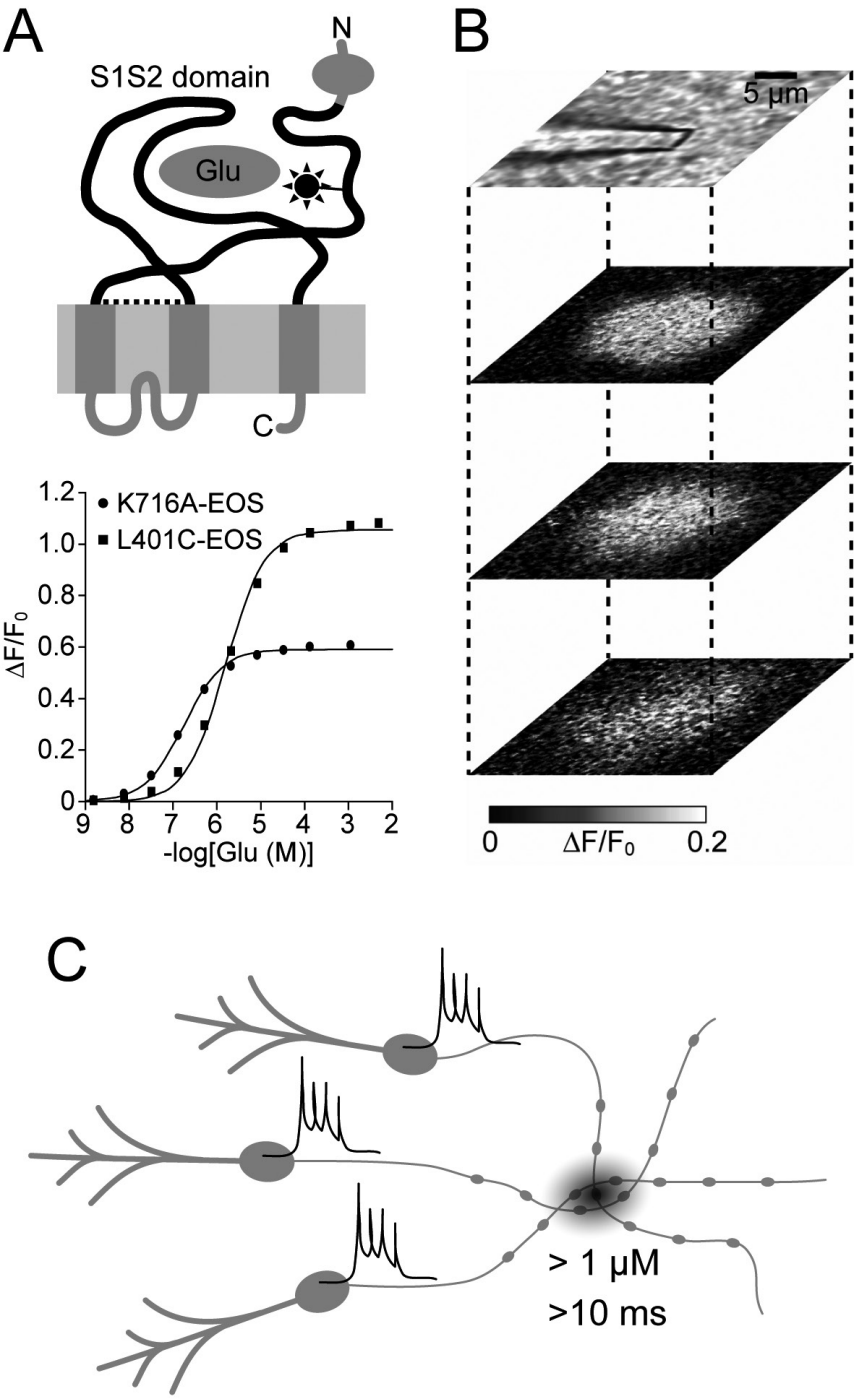
Imaging glutamate spillover with EOS. A: (Top) Structure of EOS. Glutamate binding domain (S1S2) of GluA2 subunit was isolated and the fluorophore was labeled. (Bottom) Dose-response relationship of fluorescence intensity of EOS against glutamate. B: Increases in fluorescence intensity of EOS in response to afferent stimulation in the acute cerebellar slice. C: Spatio-temporal cluster of glutamate release induces a local increase in extrasynaptic glutamate concentration. Modified from Okubo *Proc Natl Acad Sci U S A* 2010 and Okubo *Folia Pharmacol Jpn* 2014.

To image glutamate dynamics, a fluorescent glutamate indicator named Glutamate (E) Optical Sensor (EOS) was developed^9, 16)^ (Figure 1). EOS is a hybrid type indicator consisting of a glutamate binding protein and a small-molecule fluorophore. The extracellular domain of the glutamate receptor served as the glutamate binding protein, and the small-molecule fluorophore was attached near the binding pocket (Figure 1A). Changes in the fluorescence signal occur upon glutamate binding because of the environmental sensitivity of the fluorophore component. By screening of candidates, several indicators with high affinity and large signal amplitude were obtained (Figure 1A).

**Figure 2:**
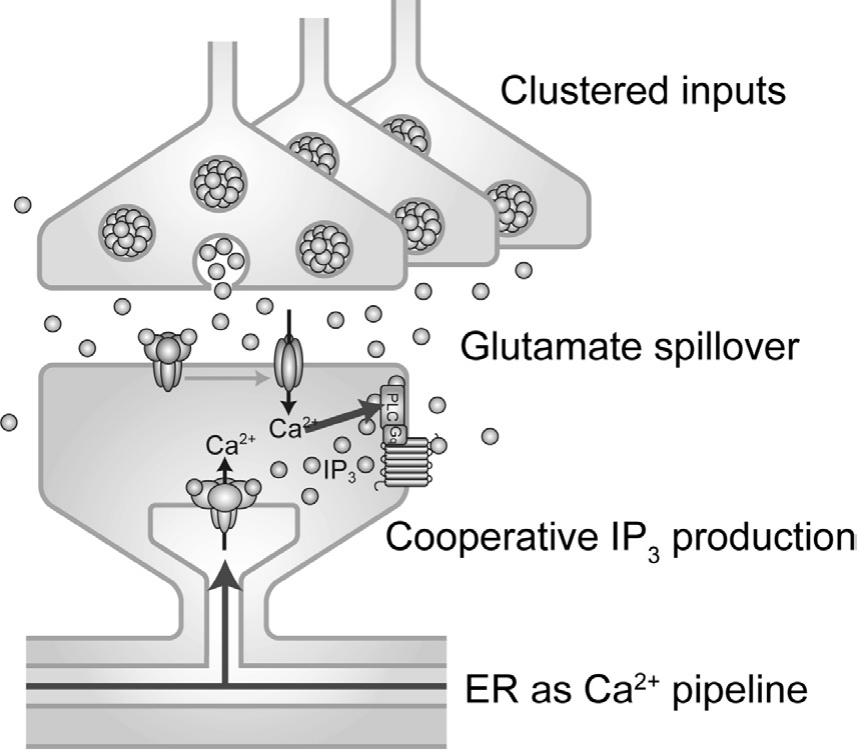
Synaptic mGluR signaling mechanisms revealed by fluorescence imaging. Glutamate spillover induced by spatio-temporal cluster of inputs forms the unit of synaptic mGluR signaling. Crosstalk between mGluR and AMPAR in IP_3_ production is a physiological booster of synaptic mGluR signaling. The ER functions as a Ca^2+^ store for the rapid and efficient redistribution of Ca^2+^ upon synaptic mGluR signaling. Modified from Okubo *Folia Pharmacol Jpn* 2014.

EOS labels extrasynaptic sites in acute brain slices and the brain *in vivo*. Changes in EOS fluorescence induced by glutamate spillover were imaged by two-photon microscopy. In response to synaptic inputs, local increases in extrasynaptic glutamate concentration were detected (Figure 1B). To induce detectable glutamate spillover, repetitive inputs of high frequency (50-100 Hz) were necessary. Furthermore, while stimulation of a single axon was insufficient to induce glutamate spillover, stimulation of an axon bundle locally induced glutamate spillover that was sufficient to activate mGluRs. These results indicate that the spatial and temporal summation of glutamate release is essential for the glutamate spillover to activate mGluRs (Figure 1C). Therefore, not a single synaptic event, but a spatio-temporal cluster of glutamate release events is the unit of synaptic mGluR signaling (Figure 2).

## Cooperative production of IP_3_

During mGluR activation, ionotropic glutamate receptors, such as the *α*-amino-3-hydroxy-5-methyl-4-isoxazolepropionic acid receptor (AMPAR), expressed within the synaptic cleft, are also activated. However, it was unclear how this simultaneous activation of different types of glutamate receptors affected mGluR-IP_3_-Ca^2+^ signaling at synapses. I conjectured that IP_3_ might play a key role in the potential crosstalk between mGluRs and AMPARs.

**Figure 3:**
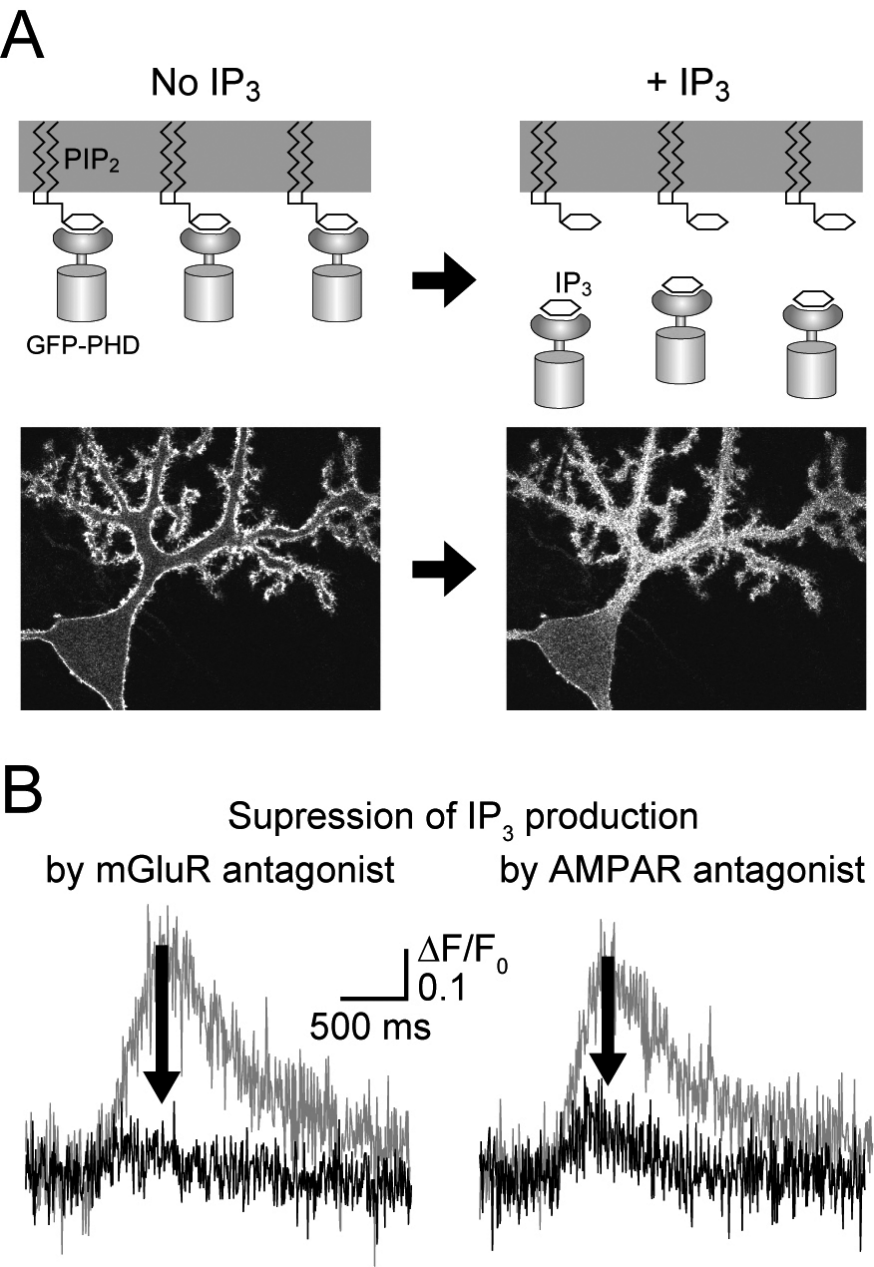
Imaging IP_3_ production with GFP-PHD. A: Schematic of IP_3_ imaging by GFP-PHD. GFP-PHD translocates from the plasma membrane to the cytosol upon IP_3_ production. B: IP_3_ production cooperatively mediated by mGluR and AMPAR. Both mGluR and AMPAR antagonists blocked synaptic IP_3_ production. Modified from Okubo *J Neurosci* 2004 and Okubo *Antioxid Redox Signal* 2011.

IP_3_ was imaged with GFP-PHD, a genetically encoded IP_3_ indicator^12, 13, 17)^ (Figure 3A). GFP-PHD is a fusion protein of green fluorescent protein (GFP) and the pleckstrin homology (PH) domain. The PH domain binds to phosphatidylinositol 4,5- bisphosphate (PIP_2_) in the plasma membrane. The PH domain also binds to IP_3_ with 20-fold higher affinity. Therefore, GFP-PHD translocates from the plasma membrane to the cytosol in response to increases in IP_3_ concentration (Figure 3A). By imaging this translocation of GFP-PHD, we can analyze IP_3_ dynamics.

GFP-PHD was expressed in a neuron in acute brain slices using a viral vector and imaged with two-photon microscopy. Translocation of GFP-PHD was monitored by the increase in fluorescence intensity in the cytosol of fine dendrites. IP_3_ production was observed upon the repetitive stimulation of axon bundles, consistent with a requirement for glutamate spillover. This IP_3_ production was blocked by an mGluR antagonist, as expected (Figure 3B). Surprisingly, an AMPAR antagonist also significantly blocked IP_3_ production (Figure 3B). These results indicate a cooperative contribution of mGluR and AMPAR to IP_3_ production. I then investigated the cellular components mediating this cooperativity and found that intracellular loading with a G protein inhibitor and a Ca^2+^ chelator blocked IP_3_ production. This strongly suggests that AMPAR-dependent Ca^2+^ influx via voltage-gated Ca^2+^ channels enhances mGluR-G protein-dependent IP_3_ production (Figure 2). This crosstalk between mGluR and AMPAR can function as a physiological booster of IP_3_ signaling at synapses.

## Diffusion-dependent refilling of Ca^2+^ in the ER

**Figure 4:**
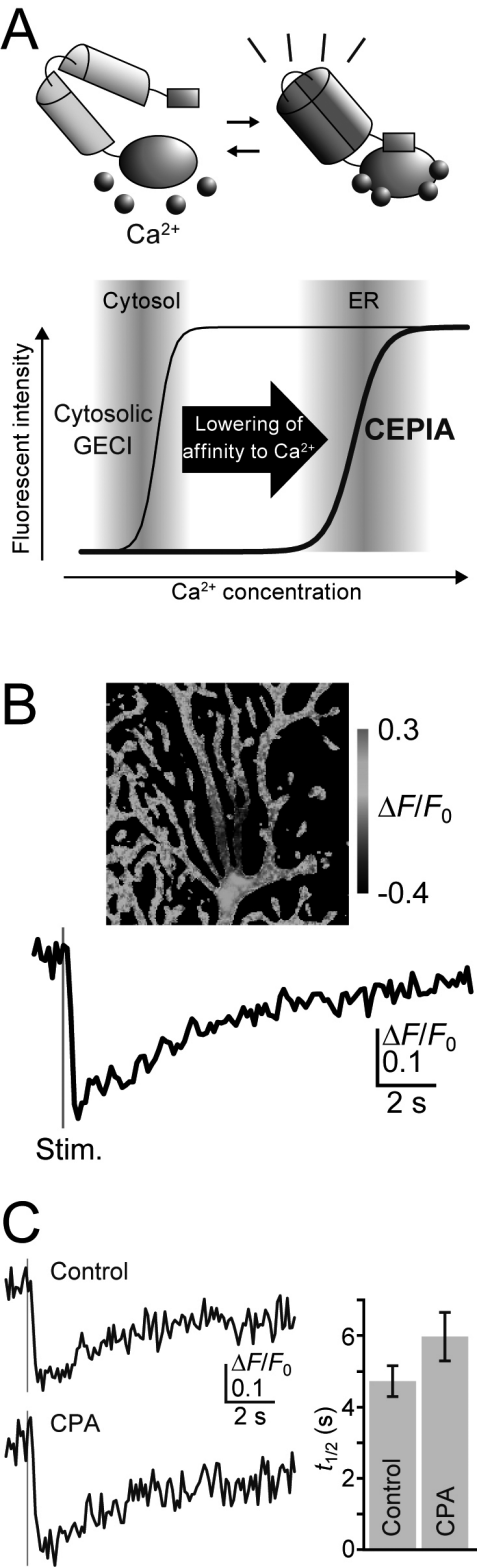
Imaging Ca^2+^ dynamics within the ER with CEPIA. A: Ca^2+^ affinity of CEPIA was optimized for Ca^2+^ concentrations within the ER. B: Plot of G-CEPIA1*er* fluorescence intensity and corresponding grayscale image upon afferent stimulation indicate local ER Ca^2+^ depletion followed by refilling upon synaptic mGluR signaling in dendrites of a neuron. C: ER Ca^2+^ refilling mediated by lateral diffusion of Ca^2+^ within the ER network. A SERCA inhibitor (cyclopiazonic acid, CPA) had a negligible effect on recovery dynamics of ER Ca^2+^ concentration. Modified from Okubo *J Neurosci* 2015.

Activation of mGluRs induces Ca^2+^ release from the ER via IP_3_ receptors. Because the ER has a relatively small volume and limited Ca^2+^ buffering capacity compared with the cytosol at synapses, Ca^2+^ release inevitably results in the depletion of Ca^2+^ within the ER. Therefore, ER Ca^2+^ refilling mechanisms are a critical determinant of the interval of synaptic mGluR signaling. However, ER Ca^2+^ refilling mechanisms at synapses remained elusive due to a lack of indicators for imaging intraluminal Ca^2+^ dynamics within the ER. To resolve this problem, novel genetically encoded Ca^2+^ indicators (GECIs) termed calcium-measuring organelle-entrapped protein indicators (CEPIAs) were developed^15, 18)^ (Figure 4A). The Ca^2+^ concentration within the ER can reach millimolar amounts. Therefore, GECIs for cytosolic Ca^2+^ cannot be applied, and indicators with appropriately low affinity for Ca^2+^ are required. Through the mutagenesis of previously developed cytosolic GECIs, indicators that cover the ER Ca^2+^ concentration range were obtained (Figure 4A). The brightness and large signal amplitude of the new CEPIA enabled unprecedented high-resolution imaging.

G-CEPIA1*er* was expressed in neurons in acute brain slices and imaged with two-photon microscopy. G-CEPIA1*er* was observed throughout the dendrites and spines, consistent with the distribution of the ER network in neurons. Transient decreases in G-CEPIA1*er* fluorescence were observed in an input-specific manner (Figure 4B). This suggested local ER Ca^2+^ depletion mediated by mGluR signaling followed by ER Ca^2+^ refilling. The conventional view of refilling mechanisms posits a major contribution to Ca^2+^ reuptake by sarco/endoplasmic reticulum Ca^2+^-ATPase (SERCA). However, lateral diffusion of Ca^2+^ within the contiguous ER network may also have an important role, especially given the local depletion within the small dendritic compartment. Indeed, diffusional dynamics was observed in G-CEPIA1*er* responses. Responses become smaller and slower at the peripheral regions. This strongly suggests lateral diffusion of Ca^2+^ from the peripheral region to the central Ca^2+^-depleted region through the lumen of the ER. The importance of diffusion-dependent refilling was confirmed by the negligible impact of the SERCA inhibitor on recovery dynamics of ER Ca^2+^ (Figure 4C). This diffusion-dependent refilling can recover Ca^2+^ concentration within an approximately 10-second time scale after mGluR-dependent Ca^2+^ depletion. Therefore, these findings suggest that the ER functions as a Ca^2+^ store that rapidly and efficiently redistributes Ca^2+^ throughout dendrites and spines (Figure 2).

## Concluding remarks

Fluorescence imaging of mGluR signaling components revealed a clustered synaptic input as the unit of mGluR signaling, cooperative IP_3_ production as a booster of mGluR signaling and the ER as Ca^2+^ pipeline (Figure 2). By introducing my work on the fluorescence imaging of mGluR signaling, I intended to highlight the following points. First, spatio-temporal dynamics at various levels is essential for brain functions. Second, to investigate dynamics in the brain, fluorescence imaging is a powerful and indispensable tool. Third, the development of fluorescent probes and specialized optics have greatly advanced neuroscience. However, it should be emphasized that what we are capable of observing now is just the tip of the iceberg. Novel optical techniques currently in development will lead to the further discovery of novel dynamic processes and help decipher the complex and intricate mechanisms of brain functions.

## Funding

Work introduced in this review was supported by the Japan Society for the Promotion of Science KAKENHI (18790176, 20790202, 23689015, 16K08543 and 19K06936), and by grants from the Pharmacological Research Foundation, the Tokyo Society of Medical Sciences, and Brain Science Foundation.

## Author contributions

YO performed the experiments for work introduced in this review. YO wrote the manuscript. YO approved the final manuscript.

## Conflicts of Interest statement

The author declares that there are no conflicts of interest.
